# Auricular squamous cell carcinoma

**DOI:** 10.1186/1865-1380-4-50

**Published:** 2011-08-08

**Authors:** Brian Kloss, Jeff Lapoint, Katherine Dougher

**Affiliations:** 1 SUNY Upstate Medical University, Department of Emergency Medicine, NY, USA; 2 NYC Poison Control/Bellevue Hospital, Department of Emergency Medicine, NY, USA; 3 SUNY Upstate Medical University, NY, USA

## Abstract

Squamous cell carcinoma is a common cancer to the head and neck region that is typically diagnosed when it is 2 cm in size. This case report illustrates a patient who had neglected an auricular carcinoma for over a year. At the time of presentation the entire ear was infected with pseudomonas and yeast and chronic friability and bleeding caused an anemia which required blood transfusion.

## Auricular squamous cell carcinoma

A 58-year-old gentleman presented to the ED complaining of a foul-smelling, friable mass close to his right ear. It had been enlarging for the past year, and he denied fevers, night sweats or weight loss. He lives alone, is divorced and works as a farmer.

**Figure 1:**
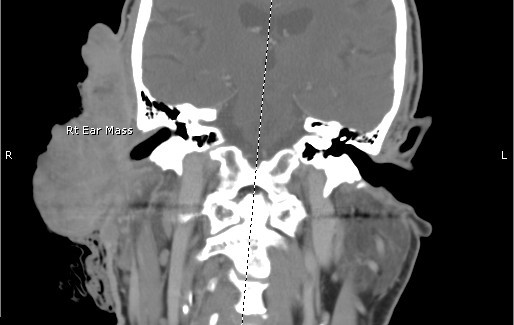
**Ear Coronal**.

**Figure 2:**
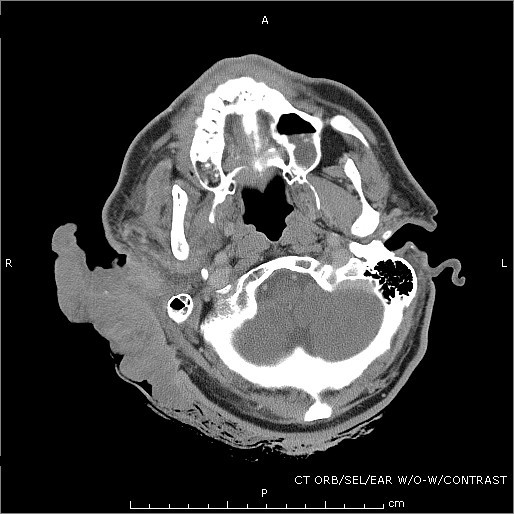
**Ear cross section**.

**Figure 3:**
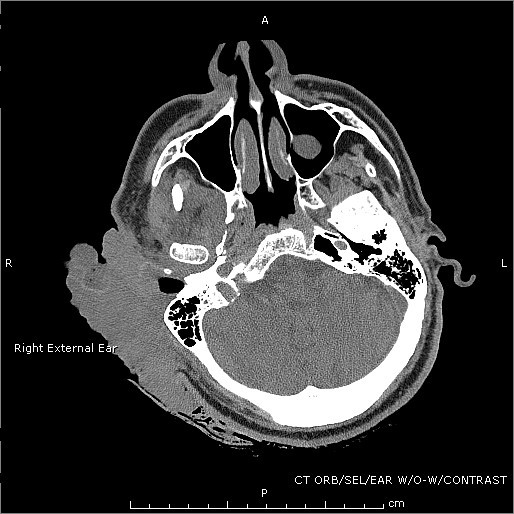
**Ear cross section**.

**Figure 4:**
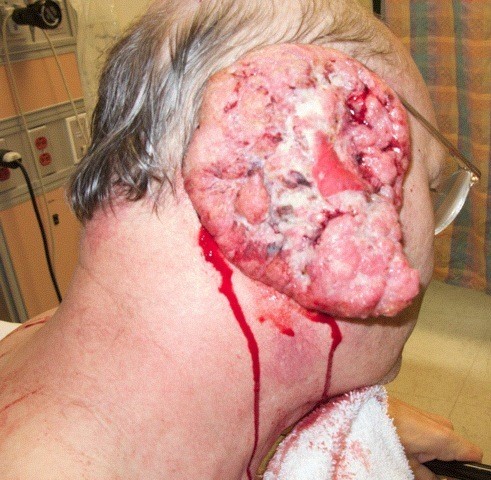
**External ear**.

Vital signs were as follows: blood pressure 190/88 mmHg, heart rate 132 bpm, respiratory rate of 20 bpm and temperature of 38.1°C. On physical exam, there was an obvious bloody and purulent mass on the right lateral aspect of his head measuring 10 × 6 cm. There was moderate cervical lymphadenopathy in the right anterior chain while the remainder of his exam was relatively benign. The patient was anemic with a hemoglobin and hematocrit of 7 and 21.5%, respectively. His white blood cell count was 10.7. CT scans were obtained, the patient was admitted, IV antibiotics were started, and the mass was ultimately removed surgically (see Figures 1, 2, 3 and 4). The mass was found to be squamous cell carcinoma with concurrent yeast and pseudomonas infection; fortunately, there was no metastasis.

Clayman et al. found that 87% of squamous cell carcinomas occur in the head and neck region with 18% of those occurring on the auricle. The average size at the time of diagnosis was 2.0 cm with the largest carcinoma measuring 17.0 cm [1]. In another study of patients with squamous cell carcinoma involving the external canal and middle ear, 1% had metastases and 87% had bony erosion at the time of diagnosis [2].

## Consent

The patient provided a written consent for photograph and publication.

## Competing interests

The authors declare that they have no competing interests.

## Authors' contributions

BK wrote the patient presentation and edited the CT images. JL obtained the photograph and patient consent. KD researched the disease process and wrote about the disease process.
